# Synthesis of Novel Pyrazinamide Derivatives Based on 3-Chloropyrazine-2-carboxamide and Their Antimicrobial Evaluation

**DOI:** 10.3390/molecules22020223

**Published:** 2017-02-02

**Authors:** Ondrej Jandourek, Marek Tauchman, Pavla Paterova, Klara Konecna, Lucie Navratilova, Vladimir Kubicek, Ondrej Holas, Jan Zitko, Martin Dolezal

**Affiliations:** 1 Department of Biological and Medical Sciences, Teaching and Research Center of Charles University, Faculty of Pharmacy in Hradec Kralove, Charles University, Zborovska 2089, Hradec Kralove 50003, Czech Republic; konecna@faf.cuni.cz; 2 Department of Pharmaceutical Chemistry and Pharmaceutical Analysis, Faculty of Pharmacy in Hradec Kralove, Charles University, Akademika Heyrovskeho 1203/8, Hradec Kralove 50005, Czech Republic; tauchmam@faf.cuni.cz (M.T.); zitkj3aa@faf.cuni.cz (J.Z.); dolezalm@faf.cuni.cz (M.D.); 3 Department of Clinical Microbiology, University Hospital Hradec Kralove, Sokolska 581, Hradec Kralove 50005, Czech Republic; pavla.paterova@fnhk.cz; 4 Department of Pharmacology and Toxicology, Faculty of Pharmacy in Hradec Kralove, Charles University, Akademika Heyrovskeho 1203/8, Hradec Kralove 50005, Czech Republic; navratl2@faf.cuni.cz; 5 Department of Biophysics and Physical Chemistry, Faculty of Pharmacy in Hradec Kralove, Charles University, Akademika Heyrovskeho 1203/8, Hradec Kralove 50005, Czech Republic; kubicek@faf.cuni.cz; 6 Department of Pharmaceutical Technology, Faculty of Pharmacy in Hradec Kralove, Charles University, Akademika Heyrovskeho 1203/8, Hradec Kralove 50005, Czech Republic; holao3aa@faf.cuni.cz

**Keywords:** tuberculosis, pyrazinamide, microwave-assisted, cytotoxicity, antibacterials, antifungals, benzylamines

## Abstract

Aminodehalogenation of 3-chloropyrazine-2-carboxamide with variously substituted benzylamines yielded a series of fifteen 3-benzylaminopyrazine-2-carboxamides. Four compounds possessed in vitro whole cell activity against *Mycobacterium tuberculosis* H37R_v_ that was at least equivalent to that of the standard pyrazinamide. MIC values ranged from 6 to 42 µM. The best MIC (6 µM) was displayed by 3-[(4-methylbenzyl)amino]pyrazine-2-carboxamide (**8**) that also showed low cytotoxicity in the HepG2 cell line (IC_50_ ≥ 250 µM). Only moderate activity against *Enterococcus faecalis* and *Staphylococcus aureus* was observed. No activity was detected against any of tested fungal strains. Molecular docking with mycobacterial enoyl-ACP reductase (InhA) was performed to investigate the possible target of the prepared compounds. Active compounds shared common binding interactions of known InhA inhibitors. Antimycobacterial activity of the title compounds was compared to the previously published benzylamino-substituted pyrazines with differing substitution on the pyrazine core (carbonitrile moiety). The title series possessed comparable activity and lower cytotoxicity than molecules containing a carbonitrile group on the pyrazine ring.

## 1. Introduction

Tuberculosis (TB) still remains a global health problem despite the decreasing total incidence of new cases. It is the second leading cause of death in the group of infectious diseases [1]. It is mainly caused by *Mycobacterium tuberculosis* (*M. tbc*). An estimated 10.4 million people developed TB and 1.8 million people died from TB in 2015 [1]. Although huge progress was noted in diagnostics and treatment, a major problem has arisen with resistance of mycobacterial strains to current therapies. These multidrug-resistant (MDR-TB) or extensively drug-resistant (XDR-TB) strains are immense threat that is necessary to focus on. A partial headway was made with the discovery of bedaquiline, a novel drug active against resistant mycobacterial strains [2].

The current common therapy for non-resistant TB is based on administering a cocktail of first-line antitubercular agents (rifampicin/rifabutin, isoniazid, ethambutol and pyrazinamide) within a period lasting from six to twelve months. The last mentioned drug—pyrazinamide (PZA)—is successfully used to shorten the time needed for treatment up to two thirds. It is characterized by its unique ability to kill dormant forms of mycobacteria [3]. PZA is endowed with multiple mechanisms of action based on its parent form or its metabolite pyrazinoic acid (POA). One of the first theories associated with the mechanism of action of PZA is based on active transport of PZA into the mycobacterial cell and its subsequent activation via nicotinamidase (pyrazinamidase; EC 3.5.1.19) to POA. Upon intracellular accumulation, POA acidifies the inner compartment of this cell causing a disruption of membrane potentials and a dysfunction of pH-sensitive enzymes [4]. This principle was disproved last year by Peterson et al. [5]. Both PZA and POA also inhibit the specific mycobacterial enzyme Fatty Acid Synthase I (FAS I; EC 2.3.1.85). Inhibition of FAS I leads to a depletion of mycolic acids and to the disruption of a mycobacterial cell wall function [6]. Further research indicated that POA also inhibits *trans*-translation. This process is also vital for cells’ survival and its blockade induces cellular death [7]. One of the latest studies revealed that PZA inhibits aspartate decarboxylase (PanD; EC 4.1.1.11). This enzyme is responsible for the conversion of L-aspartic acid to β-alanine, which is necessary for CoA synthesis. A blockade of this biochemical pathway usually leads to the energy depletion and to the inability to survive [8].

**Figure 1:**
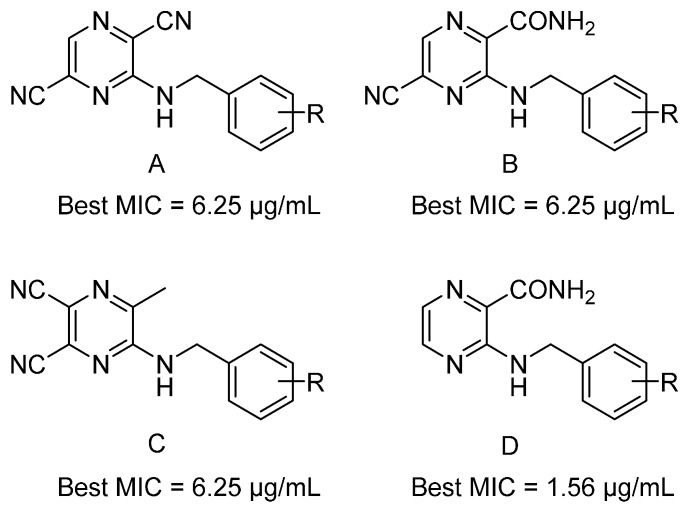
General structures of discussed compounds (A, B—Zitko et al. [16]; C—Jandourek et al. [17]; D—this work) and best MICs against *M. tbc* H37R_v_.

PZA itself evinces unique physicochemical and chemical properties and its small molecule is exceedingly suitable for modifications. Alkylamino substitution of the pyrazine ring in positions 5- and 6- is quite well known and often results in good antimycobacterial activities [9,10,11,12]. This work deals with benzylamino derivatives since compounds containing a benzylamino moiety were previously reported as anti-infective substances. *N*-Benzylpyrazine-2-carboxamides (i.e., containing a benzylamino moiety attached to the pyrazine nucleus via a carbonyl linker) were reported by Servusova et al. to show moderate antitubercular activities [13,14,15]. Benzylamino derivatives of pyrazine (with a benzylamino moiety attached directly to the pyrazine core) reported by Zitko et al. and Jandourek et al. showed moderate activities as well [16,17]. In contrast to the compounds proposed in this article, the previously reported benzylamino derivatives contained at least one carbonitrile moiety (see Figure 1).

Such modifications did not lead to an improved antitubercular effect. These results confirmed the validity of our intention to prepare novel derivatives with different substituents to improve efficacy and safety.

We have chosen the pyrazine ring without carbonitrile moieties (lower cytotoxicity), with a free carboxamide group (possibility of activation to POA) and a benzylamino substituent directly attached through its amino group to the pyrazine core. These compounds were investigated to establish whether this combination made on the basis of previous publications would demonstrate better biological properties.

3-Chloropyrazine-2-carboxamide was allowed to react with variously substituted benzylamines by means of the nucleophilic substitution of chlorine. Some of the intended reactions were carried out using microwave-assisted synthesis, which can save time and used materials according to the literature. In addition, yields usually tend to be higher [18]. We have investigated the differences between conventional organic synthesis (conventional heating methods) and microwave-assisted reaction and a comparison is reported.

Additional biological assays (antimycobacterial, antibacterial, antifungal) were performed to establish the scope of activity of these novel pyrazinamide derivatives that were preliminarily published in electronic conference contributions [19,20].

Finally, prepared compounds were docked into the active site of *M. tuberculosis* enoyl-ACP reductase (InhA; EC 1.3.1.9). InhA has been repeatedly evaluated as an effective antimycobacterial target. It is a crucial enzyme involved in the biosynthesis of long chain fatty acids. It is responsible for the reduction of the double bond between C2 and C3 of the enoyl intermediate linked to the acyl carrier protein (ACP). The inhibition of InhA leads to an insufficient synthesis of mycolic acids, which are essential for mycobacterial cell wall integrity [21]. A molecular modeling study has been performed to verify if InhA inhibition is a possible mechanism of action for the prepared derivatives due to their structural similarity to known InhA inhibitors such as triclosan or PT70. It is obvious that the requirement of two planar heteroaromatic or aromatic structural fragments connected with a linker, which is specifically represented by -NH-CH_2_- formation, is fulfilled. Another resemblance is actually expressed by a carbonyl part of carboxamide group serving as a hydrogen bond accepting moiety. These structural features are shared with typical InhA inhibitors [22]. Molecular targets of PZA/POA as discussed above were considered for docking simulations. However, there are limitations that make them inapplicable for our compounds. The complex of POA bound to the ribosomal protein S1 (involved in *trans*-translation process) was resolved (pdb: 4NNI). From this complex it is obvious that POA could be substituted in positions 5- and/or 6-, but not in position 3- [23]. The pyrazinamidase (PncA) active site cavity is quite small and from a model based on the X-ray determined structure of PncA (pdb: 3PL1), it is quite obvious that the only position for possible substitutions of PZA is position 6- of the pyrazine ring [24]. Substitution of position 3- by a large moiety (benzylamino in our case) would push the PZA core away from the position and orientation needed for an enzymatic conversion by PncA. The crystal structure of aspartate decarboxylase (PanD) was resolved (pdb: 2C45), but there are no complexes with bound PZA/POA. The binding site and interaction patterns for PZA/POA are therefore unknown. Simulations of PZA/POA binding to PanD by docking and docking followed by molecular dynamics simulation of the complex gave uncertain results [25]. The FAS I enzyme complex is very extensive so an X-ray resolved crystal structure of sufficient quality is not yet available [26].

## 2. Results and Discussion

### 2.1. Chemistry

The starting compound 3-chloropyrazine-2-carboxamide was synthesized from 3-chloro-pyrazine-2-carbonitrile through a partial hydrolysis of the nitrile group under controlled conditions involving a specific pH and temperature [27]. This procedure was chosen on the basis of the higher yields obtained compared to the direct amidation of the pyrazine ring [27,28].

**Scheme 1:**
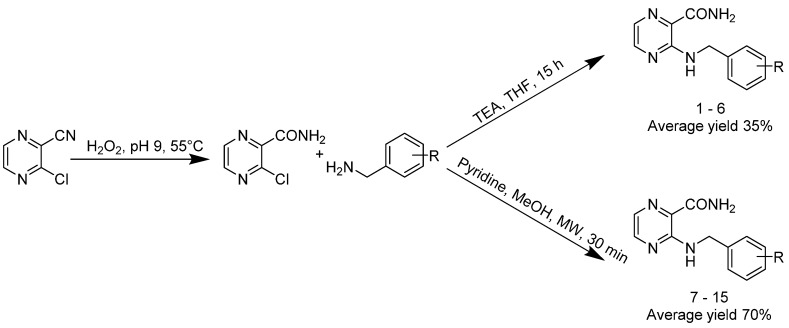
Synthesis of the starting compound 3-chloropyrazine-2-carboxamide and its amino-dehalogenation reactions with variously substituted benzylamines carried out under different conditions.

This initial compound then reacted with variously substituted benzylamines (see Scheme 1). Six final compounds **1**–**6** were prepared by conventional heating methods utilizing tetrahydrofuran (THF) as a solvent, triethylamine as a base and two equivalents of corresponding benzylamine. Reactions were stopped after 15 h and their progress was checked using TLC and the hexane−ethyl acetate (1:1) system as eluent. Products were purified by preparative flash chromatography and the final yields ranged between 24% and 50%. These low values prompted us to complete the series under microwave conditions. Thus compounds **7**–**15** were prepared successfully in a MW reactor using methanol as a solvent and pyridine as a base. Sealed thick-walled tubes were used as an over-pressurized system to reach a higher temperature (140 °C) than the standard boiling point of methanol. Time needed for a conversion was shortened from 15 h to 30 min. The final compounds were also purified using flash chromatography. Not surprisingly, the yields were 70% on average, and ranged from 26% to 80%. The lowest yield (26%) was observed for compound **15** that is substituted with a nitro group in the *meta* position. In general, it is hard to obtain higher yields with this kind of substitution as its electron withdrawing properties decrease the nucleophilicity of the benzylamino nitrogen. The difference between these two approaches can highlight the advantages of microwave-assisted reactions. The time needed to complete conversions was shortened ten times and the amount of solvents used was substantially minimized. The variance between yields was also significant. On the other hand, some limitations are emerging with this technology. It can be stated that not every base is usable for microwave-assisted reactions. Triethylamine, which acted as a base for conventional heating reactions, is inapplicable for MW in consideration of the fact that a molecule of TEA decomposes during the process. Byproducts (e.g., diethylamine) can react with starting compounds giving inadvertent side products [29,30].

The final products were characterized by ^1^H- and ^13^C-NMR spectra, IR spectroscopy, elemental analysis, and also by their melting points. The acquired analytical data were fully in accordance with proposed structures.

### 2.2. Biological Assays

#### 2.2.1. Antimycobacterial Screening

All of prepared compounds were screened for in vitro activity against *Mycobacterium tuberculosis* H37R_v_, *M. kansasii* My 235/80 and *M. avium* 152/73. Experiments were carried out in a liquid broth with the pH value adjusted to 5.6. It is known that in vitro activity of PZA is strongly dependent on pH (MIC values ranging from units of µg/mL at pH 5.5 to hundreds of µg/mL in neutral conditions) [31,32,33,34]. Using neutral pH and high concentration is not appropriate for our compounds due to their limited solubility in the water-based testing medium. We were aware that acidic pH can negatively affect the growth of *M. tbc*. However, it was proved that *M. tbc* can be cultivated for 14–21 days of incubation under these conditions [35,36,37,38]. In our experiments, drug-free controls were included to validate the growth of mycobacteria under chosen acidic conditions. A microtitration plate assay was used to determine the activity and results were read using a resazurin dye-based methodology.

**Figure 2:**
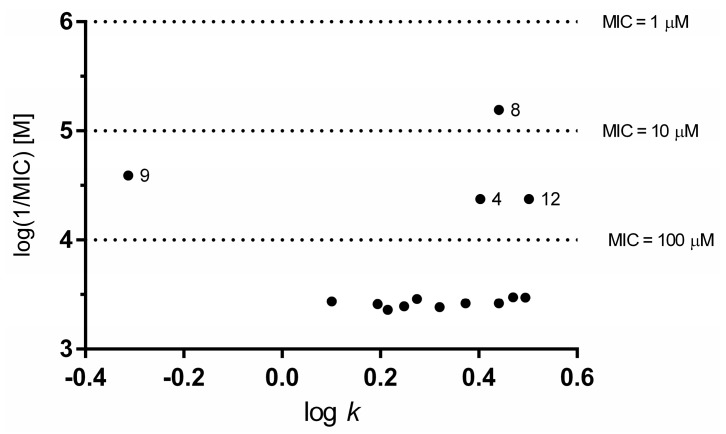
Dependency of antimycobacterial activity (1/MIC) on experimental parameter of lipophilicity log*k*.

The series contains compounds that showed activity against *M. tuberculosis* H37R_v_. Such activity was detected for compounds **4**, **8**, **9** and **12**, with MIC values in the range 1.56–12.5 µg/mL (resp. 6–42 µM) as presented in Table 1. It cannot be stated that there is a dependence between the lipophilicity and the activity. The values of lipophilicity either for active or inactive compounds vary over a wide range (see Figure 2). Compounds **4** and **12** are substituted with a trifluoromethyl group, which is usually used fruitfully to prepare potentially active substances. On the other hand, these structures often show an undesirable higher cytotoxicity [16,39,40,41]. Cytotoxicity screening was performed for that reason. Contrarily, compounds **8** and **9** are substituted with less burdening groups and the activities demonstrated by them are more auspicious.

Derivative **8** showed the best activity, which was close to the activity of the standard isoniazid (MIC = 3 µM) (see Table 1). Results were compared to the previously prepared corresponding 3-benzylamino-5-cyanopyrazine-2-carboxamides (Table 2, A), 3-benzylaminopyrazine-2,5-dicarbonitriles (Table 2, B) and 5-benzylamino-6-methylpyrazine-2,3-dicarbonitriles (Table 2, C) in order to compare the effect of cyano vs. carboxamide substitution. 

These results were mostly comparable. Nevertheless, there are some compounds that should be pointed out. Compound **4** (R = 3-CF_3_; MIC = 12.5 µg/mL) was more active than the matching substances **4-A** (MIC = 25 µg/mL) or **4-B** (MIC ≥ 100 µg/mL) and the activity of derivative **4-C** (MIC = 12.5 µg/mL) was nearly the same [16,17]. Compound **9** (R = 4-NH_2_; MIC = 6.25 µg/mL) showed better activity than **9-A** (MIC = 12.5 µg/mL), **9-B** (MIC = 25 µg/mL) or **9-C** (MIC = 25 µg/mL) [16,17]. Compound **1** (R = H; MIC ≥ 100 µg/mL) proved to be much less active than the corresponding dicarbonitriles **1-B** (MIC = 12.5 µg/mL) and **1-C** (MIC = 25 µg/mL). However, the corresponding cyanopyrazine-2-carboxamide (**1-A**; MIC ≥ 100 µg/mL) was also inactive [16,17]. In contrast, compound **8** (R = 4-CH_3_; MIC = 1.56 µg/mL) was certainly more active than the related compounds **8-B** (MIC = 6.25 µg/mL), **8-A** (MIC = 12.5 µg/mL) and **8-C** (MIC = 25 µg/mL) [16,17]. It can be deduced that carbonitrile group on the pyrazine core is not necessary to obtain better activity and compounds without this modification are equally or more active against *M. tuberculosis* (see Table 2). Compounds **8** and **9** were afterwards tested for their antimycobacterial activity against four MDR-TB strains and one XDR-TB strain. Results of this screening are listed in Table 3. 4-Amino derivative (MIC = 62.5 µM) showed better activity than 4-methyl derivative (MIC = 250 µM).

Just one compound was effective against a mycobacterial strain other than *M. tuberculosis* with activity comparable to a standard. Specifically, derivative **4** showed activity against *Mycobacterium kansasii* (MIC = 25 µg/mL, resp. 84 µM) whilst the MIC of isoniazid ranged between 1.56–12.5 µg/mL (resp. 11–91 µM).

#### 2.2.2. *Mycobacterium smegmatis* Screening

Screening was performed again on the basis of adjusted microtitration plate assay with resazurin dye staining. This complementary test was completed in order to find any relationships between activities against typical slow growing mycobacterial strains and *Mycobacterium smegmatis*. The behavior of the last named microorganism is similar to that of other strains but it is not a human pathogen. Its generation time is also advantageous because it is disproportionally shorter. The results of this assay were dissimilar to the previous antimycobacterial screening. Poor activity was observed for three compounds, namely compounds **4**, **5** and **9**, as seen in Table 1. The value 250 µg/mL was not absolutely comparable to the used standards isoniazid (15.63 µg/mL), rifampicin (0.39 µg/mL) and ciprofloxacin (0.10 µg/mL). It is thus apparent that the prepared substances are active against *M. tuberculosis* and probably act through a pathway that is not common to both types of mycobacteria.

#### 2.2.3. Antibacterial and Antifungal Screening

All compounds were tested in vitro for their activity against eight common bacterial strains and eight fungal stems using standard methodology. Four of the fifteen synthesized substances exerted a moderate to low activity against *Enterococcus faecalis*, with values ranging between 62.5 µM and 125 µM (see Table 1). Compounds **4** and **5** were active against *Staphylococcus aureus* with a mean activity of 31.25 µM. All these results were insignificant in comparison with standards used in this screening. No antifungal activity was observed for any of the prepared substances.

#### 2.2.4. Cytotoxicity Assays

The most active compounds **4**, **8**, **9** and **12** were tested for their cytotoxic effects. Results of these experiments are presented as the inhibitory concentration required to decrease the viability of the cell population to 50% from the maximal viability (IC_50_) compared to 100% cell viability control. The cytotoxicity of the tested compounds was measured using the standard hepatic cell line HepG2.

The used CellTiter 96^®^ Aqueous One Solution Assay is based on a bioreduction of tetrazolium dye MTS by cells into a coloured formazan product, which is then determined colorimetrically. Reduction of this reagent is accomplished by NADPH or NADH produced in metabolically active cells. The quantity of formazan is therefore directly proportional to the number of living cells.

Compounds can be divided into two groups according to their IC_50_ values as seen in Table 4. The first group is represented by substances **4** and **12** substituted with trifluoromethyl groups. The IC_50_ value in those cases is in the order of tens of µM, indicating a relatively high cytotoxicity. This course was expected according to the characteristics of the substituent that was stated above. It could be also connected with a higher lipophilicity causing intracellular accumulation. The second group of compounds (**8**, **9**) showed encouraging results within the order of hundreds or thousands of µM.

Actual IC_50_ values for these compounds could not be determined due to their low solubility at higher concentrations, but it is at least three orders better in comparison to compounds **4** and **12**.

The selectivity index (SI) was calculated for antimycobacterial activity (against *M. tuberculosis*) and was defined as a ratio between IC_50_ and MIC (µM). Values above 10 are considered to be safe values for a potential novel drug. This condition is met by compounds **8** and **9** (see Table 4).

#### 2.2.5. Lipophilicity Determination

**Figure 3:**
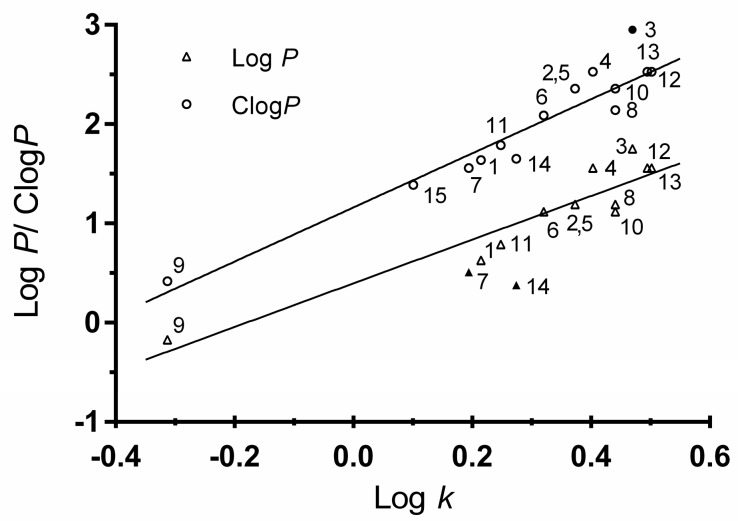
Linear dependencies of two differently calculated lipophilicity parameters on experimentally measured values.

Calculated lipophilicity parameters log*P* and Clog*P* were predicted using the ChemBioDraw Ultra 14 software (CambridgeSoft, Cambridge, MA, USA). These values were compared with log*k*, an experimentally measured lipophilicity parameter derived from retention times acquired by RP-HPLC. Theoretical (log*P*, Clog*P*) and experimental (log*k*) values were correlated to show a linear dependency, even though the values of log*P* and Clog*P* calculated by the ChemBioDraw algorithm did not reflect the influence of substituents’ positions on the aromatic ring (see Figure 3).

Correlation of these parameters can be expressed by the following regression equations. Equation (1) shows the dependency of log*P* on log*k*. Compounds **7** and **14** were excluded from the calculations because their distinct values. It could be caused by the nature of the substituent group (a methoxy group). The log*P* algorithm underestimates the lipophilicity of methoxy-substituted derivatives, especially compound **14**, according to the linear regression of log*P* on log*k*. The reason for the increased lipophilicity for compound **14** could be a formation of intramolecular *H*-bonds between the 2-OCH_3_ moiety (as an acceptor) and an amidic or amino hydrogen (donor). However, the conformational search by low mode molecular dynamics using AMBER10:EHT force field did not generate any pose with such intramolecular *H*-bond among conformers within a 7 kcal/mol energy window.

Therefore, the discrepancy of log*P* prediction for **14** should be ascribed either to a systemic error in our measurements or to a suboptimal parametrization of -OCH_3_ fragment in the algorithm:

log*P* = 0.397(±0.104) + 2.199(±0.263) log*k* R^2^ = 0.875, s = 0.194, F = 69.7, n = 12 (1)

Equation (2) expresses the dependency of Clog*P* on log*k*. This relationship is more predicative showing better regression parameters even for the methoxy derivatives **7** and **14**:

Clog*P* = 1.162(±0.074) + 2.726(±0.210) log*k* R^2^ = 0.934, s = 0.159, F = 168.6, n = 14 (2)

Compound **9** differs from the others in both parameters, which can be caused by the character of the substitution. Amino groups are usually easily ionized, which can result in distortion of the outcomes of the performed measurements.

### 2.3. Computational Studies—Docking

**Figure 4:**
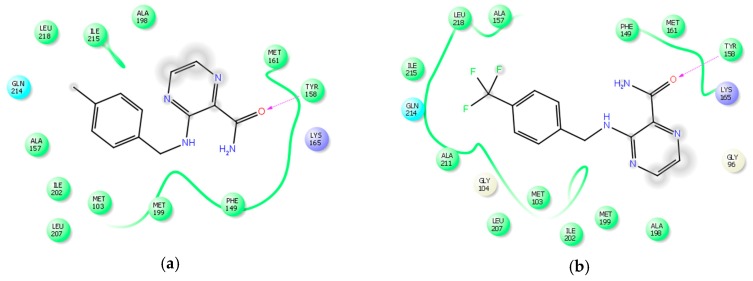
2D visualization of (**a**) compound **8** and (**b**) compound **12** in active site of InhA showing interactions with the substrate binding loop.

The affinity calculation results show the important effect of a carboxamide group in the assessed compounds. The affinity towards InhA is high within a series containing compounds with substituents in the 3-position of the pyrazine core. Such an arrangement enables close proximity of the carbonyl group to Tyr 158 and NAD^+^ ribose and allows the substituent in the aforementioned 3- position to form additional less specific interactions with substrate binding group residues at the same time (see Figure 4). 

**Figure 5:**
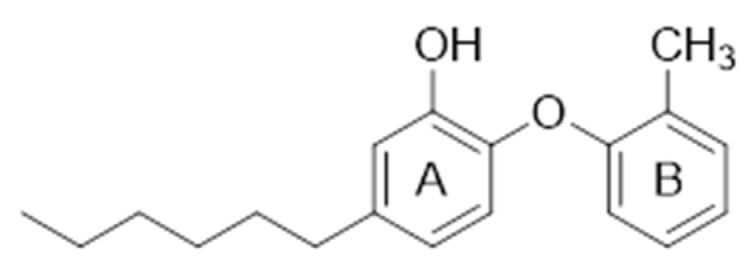
Chemical structure of PT70.

**Figure 6:**
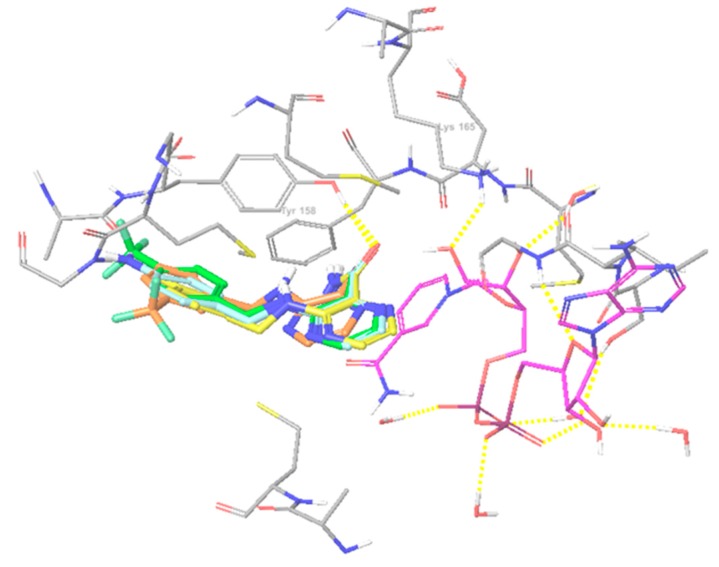
The most active compounds docked into the active site of InhA (enoyl-ACP reductase) (**4**—orange; **8**—yellow; **9**—aquamarine; **12**—green).

None of assessed compounds reached an affinity higher or equal to (3*S*)-1-cyclohexyl-*N*-(3,5-dichlorophenyl)-5-oxopyrrolidine-3-carboxamide (−9.82 kcal/mol) that is the original 4TZK ligand. The best scored compounds **4** and **12** (affinity −8.47 kcal/mol resp. −8.49 kcal/mol, see Table 5) attain similar poses within the active site as most of the direct InhA inhibitors, forming a hydrogen bond network between Tyr 158, NAD^+^ ribose and the pyrazine core. The carboxamide moiety fulfils a similar role to the carbonyl moiety of the original ligand 4TZK or a phenolate of known inhibitors such as triclosan or PT70 (ring A) (see Figure 5). The hydrophobic benzyl part of discussed compounds exerts hydrophobic interactions with lipophilic residues of the substrate binding loop (Met 103, Pro 193, Met 199, Leu 207 and Ile 215, see Figure 6). An orientation of this benzyl moiety is in accordance with an orientation of hexyl chain of PT70 that is oriented in the hydrophobic entry tunnel, which normally hosts the long alkyl chain of mycolic acid intermediate (see Figure 5).

The most efficient compound **8** did not achieve such a promising score compared to the standard ligand. This might happen because of a less hydrophobic substitution of the non-pyrazine molecule part that results in a less potent hydrophobic interactions with substrate binding loop hydrophobic residues.

## 3. Materials and Methods

### 3.1. General Methods

All chemicals were of reagent or higher grade of purity. They were mainly purchased from Sigma-Aldrich or Fluka (Sigma-Aldrich, Co., Steinheim, Germany). Silica gel 60 F_254_ plates for thin layer chromatography (TLC) were provided by Merck KGaA (Darmstadt, Germany). Microwave assisted reactions were performed using a CEM Discover focused field microwave reactor equipped with an autosampler Explorer 24 (CEM Corporation, Matthews, NC, USA). Reaction progress was monitored with CEM’s Synergy™ software. All compounds were purified using preparative flash chromatography (CombiFlash^®^ RF, Teledyne Isco, Inc., Lincoln, NE, USA). Silica gel (0.040–0.063 mm) was used as a stationary phase (Merck KGaA) and hexane (Lach-Ner, s.r.o., Neratovice, Czech Republic) together with ethyl acetate (Penta, Prague, Czech Republic) as a mobile phase in a gradient elution. NMR spectra were acquired at ambient temperature by Varian Mercury-VxBB 300 spectrometer (Varian Corp., Palo Alto, CA, USA) operating at 299.95 MHz for ^1^H and 75.43 MHz for ^13^C or Varian VNMR S500 spectrometer operating at 499.87 MHz for ^1^H, and 125.71 MHz for ^13^C. Chemical shifts were referenced in ppm (δ) and were indirectly related to TMS (tetramethylsilane) via the solvent signal (2.49 for ^1^H and 39.7 for ^13^C in DMSO-*d_6_* or 7.28 for ^1^H and 77.0 for ^13^C in CDCl_3_). Infrared spectra were recorded using the Attenuated Total Reflectance (ATR) methodology on a Nicolet 6700 FT-IR spectrometer (Nicolet–Thermo Scientific, Waltham, MA, USA) on germanium crystals. Elemental analysis was performed on an EA 1110 CHNS Analyser (Fisons Instruments S. p. A., Carlo Erba, Milano, Italy) and values were given as percentages. Melting points were determined by a SMP3 Stuart Scientific apparatus (Bibby Sterling Ltd., Staffordshire, UK) in open capillary tubes and are uncorrected. Yields are expressed as percentages and refer to isolated products after all purification steps.

### 3.2. General Synthetic Procedure

The starting compound 3-chloropyrazine-2-carboxamide was prepared via partial hydrolysis of the nitrile group of 3-chloropyrazine-2-carbonitrile (Fluorochem, Co., Hadfield, Derbyshire, UK). A mixture of concentrated (30%) hydrogen peroxide (29 mL) and water (195 mL) was prepared and alkalinized with an 8% (*w*/*v*) solution of sodium hydroxide to obtain a solution with pH 9. The carbonitrile (104 mmol) was then added portionwise into the heated (50 °C) mixture over a period of 30 min. The whole mass was stirred for an additional 2.5 h at 55 °C while the pH was periodically monitored and alternatively adjusted to the value of 9 by adding a few drops of 8% NaOH solution. The reaction mixture was cooled in a fridge to initiate crystallization. The crude product was recrystallized from ethanol [27]. The yield of this reaction was approximately 80%.

Compounds **1**–**6** were prepared according to conventional organic synthesis methods. 3‑Chloropyrazine-2-carboxamide (1.27 mmol) was dissolved in THF (20 mL) in a round bottom flask and after that treated with two equivalents of the corresponding benzylamine and an equimolar amount of triethylamine. The reaction was conducted with continuous stirring and heating (70 °C) under reflux in an oil bath for 15 h. Compounds **7**–**15** were synthesised using a microwave reactor with a focused field. 3-Chloropyrazine-2-carboxamide (1.27 mmol) was put into a thick-walled tube together with the corresponding benzylamine (2.54 mmol), pyridine (1.27 mmol), methanol (approx. 5 mL) and a magnetic stir bar and then sealed with a special cap. The reaction parameters were set according to the previously published paper as follows—140 °C, 30 min, 200 W [29]. Reaction progress was checked by TLC (hexane:ethyl acetate—1:1). Regardless of the synthesis method used, all reaction mixtures were adsorbed on silica and subjected to preparative flash chromatography (hexane and ethyl acetate, gradient elution, detection wavelengths 260 nm and 280 nm). Products were recrystallized from ethanol or ethanol and water if necessary. All final substances were chemically characterized (^1^H-NMR, ^13^C-NMR, IR, melting point and elemental analysis).

### 3.3. Analytical Data of the Prepared Compounds

*3-(Benzylamino)pyrazine-2-carboxamide* (**1**). Yellow solid. Yield 35%; M.p. 121.3–123.5 °C (125-126 °C in previously published work [42]); IR (ATR-Ge, cm^−1^): 3452 (-NH-), 3126 (-CONH_2_), 2869 (-CH_2_-), 1689 (-C=O), 1584, 1529, 1508, 1473, 1369, 1236, 1180 (arom.); ^1^H-NMR (500 MHz, DMSO) δ 9.14 (1H, t, *J* = 5.7 Hz, NH), 8.23 (1H, d, *J* = 2.4 Hz, H6), 8.18 (1H, bs, NH_2_), 7.79 (1H, d, *J* = 2,4 Hz, H5), 7.70 (1H, bs, NH_2_), 7.33−7.28 (4H, m, H2′, H3′, H5′, H6′), 7.25−7.20 (1H, m, H4′), 4.63 (2H, d, *J* = 5.7 Hz, CH_2_). ^13^C-NMR (125 MHz, DMSO) δ 169.0, 154.3, 146.5, 139.7, 130.2, 128.6, 127.4, 127.0, 126.7, 43.5. Elemental analysis found: C, 62.88%; H, 5.50%; N, 24.16%. Calculated for C_12_H_12_N_4_O (MW 228.25): C, 63.15%; H, 5.30%; N, 24.55%.

*3-[(3-Chlorobenzyl)amino]pyrazine-2-carboxamide* (**2**). Dark yellow solid. Yield 32%; M.p. 146.1–148.9 °C; IR (ATR-Ge, cm^−1^): 3435 (-NH-), 3196 (-CONH_2_), 2926 (-CH_2_-), 1666 (-C=O), 1578, 1534, 1511, 1408, 1338, 1262, 1231, 1189 (arom.); ^1^H-NMR (500 MHz, DMSO) δ 9.17 (1H, t, *J* = 6.0 Hz, NH), 8.22 (1H, d, *J* = 2.4 Hz, H6), 8.18 (1H, bs, NH_2_), 7.79 (1H, d, *J* = 2.4 Hz, H5), 7.71 (1H, bs, NH_2_), 7.39–7.23 (4H, m, H2′, H4′, H5′, H6′), 4.65 (2H, d, *J* = 6.0 Hz, CH_2_). ^13^C-NMR (125 MHz, DMSO) δ 168.9, 154.2, 146.5, 142.6, 133.2, 130.5, 130.4, 127.1, 126.9, 126.9, 126.0, 43.0. Elemental analysis found: C, 55.18%; H, 4.51%; N, 20.94%. Calculated for C_12_H_11_ClN_4_O (MW 262.69): C, 54.87%; H, 4.22%; N, 21.33%.

*3-[(3,4-Dichlorobenzyl)amino]pyrazine-2-carboxamide* (**3**). Yellow solid. Yield 24%; M.p. 169.5–172.1 °C; IR (ATR-Ge, cm^−1^): 3472 (-NH-), 3174 (-CONH_2_), 2924 (-CH_2_-), 1677 (-C=O), 1576, 1529, 1500, 1465, 1416, 1338, 1233, 1187 (arom.); ^1^H-NMR (300 MHz, DMSO) δ 9.19 (1H, t, *J* = 6.2 Hz, NH), 8.24–8.20 (1H, m, H6), 8.18 (1H, bs, NH_2_), 7.82–7.79 (1H, m, H5), 7.72 (1H, bs, NH_2_), 7.58–7.53 (1H, m, H5′), 7.53 (1H, d, *J* = 1.7 Hz, H6′), 4.64 (2H, d, *J* = 6.2 Hz, CH_2_). ^13^C-NMR (75 MHz, DMSO) δ 168.8, 154.1, 146.4, 141.4, 131.0, 130.7, 130.6, 129.3, 129.3, 127.7, 127.0, 42.5. Elemental analysis found: C, 49.09%; H, 3.76%; N, 18.69%. Calculated for C_12_H_10_Cl_2_N_4_O (MW 298.14): C, 48.86%; H, 3.39%; N, 18.86%.

*3-[(3-Trifluoromethylbenzyl)amino]pyrazine-2-carboxamide* (**4**). Dark yellow solid. Yield 31%; M.p. 98.1–100.3 °C; IR (ATR-Ge, cm^−1^): 3468 (-NH-), 3201 (-CONH_2_), 2937 (-CH_2_-), 1674 (-C=O), 1581, 1530, 1507, 1453, 1416, 1236, 1185, 1164 (arom.), 1331 (-CF_3_); ^1^H-NMR (500 MHz, DMSO) δ 9.23 (1H, t, *J* = 6.1 Hz, NH), 8.22 (1H, d, *J* = 2.2 Hz, H6), 8.19 (1H, bs, NH_2_), 7.80 (1H, d, *J* = 2.2 Hz, H5), 7.72 (1H, bs, NH_2_), 7.66 (1H, s, H2′), 7.64–7.50 (3H, m, H4′, H5′, H6′), 4.74 (2H, d, *J* = 6.1 Hz, CH_2_). ^13^C-NMR (125 MHz, DMSO) δ 168.9, 154.2, 146.4, 141.6, 131.4, 130.5, 129.6, 129.2 (q, *J* = 31.3 Hz), 127.0, 124.4 (q, *J* = 272.0 Hz), 123.8 (q, *J* = 3.9 Hz), 123.7 (q, *J* = 3.9 Hz), 43.1. Elemental analysis found: C, 52.48%; H, 3.40%; N, 18.76%. Calculated for C_13_H_11_F_3_N_4_O (MW 297.14): C, 52.71%; H, 3.74%; N, 18.91%.

*3-[(4-Chlorobenzyl)amino]pyrazine-2-carboxamide* (**5**). Yellow solid. Yield 59%; M.p. 141.0–142.0 °C; IR (ATR-Ge, cm^−1^): 3408 (-NH-), 3290 (-CONH_2_), 2930 (-CH_2_-), 1682 (-C=O), 1589, 1530, 1510, 1468, 1413, 1339, 1232, 1188 (arom.); ^1^H-NMR (500 MHz, DMSO) δ 9.17 (1H, bs, NH), 8.23–8.20 (1H, m, H6), 8.18 (1H, bs, NH_2_), 7.81–7.77 (1H, m, H5), 7.71 (1H, bs, NH_2_), 7.38–7.30 (4H, m, H2′, H3′, H5′, H6′), 4.63 (2H, d, *J* = 5.9 Hz, CH_2_). ^13^C-NMR (125 MHz, DMSO) δ 168.9, 154.2, 146.5, 139.0, 131.4, 130.4, 129.2, 128.5, 126.9, 42.8. Elemental analysis found: C, 55.08%; H, 4.64%; N, 21.58%. Calculated for C_12_H_11_ClN_4_O (MW 262.69): C, 54.87%; H, 4.22%; N, 21.33%.

*3-[(2-Methylbenzyl)amino]pyrazine-2-carboxamide* (**6**). Yellow solid. Yield 38%; M.p. 148.3–150.5 °C; IR (ATR-Ge, cm^−1^): 3435 (-NH-), 3201 (-CONH_2_), 2925 (-CH_2_-), 1665 (-C=O), 1575, 1536, 1512, 1465, 1411, 1339, 1229, 1192 (arom.); ^1^H-NMR (500 MHz, DMSO) δ 9.03 (1H, bs, NH), 8.24 (1H, s, H6), 8.19 (1H, bs, NH_2_), 7.79 (1H, s, H5), 7.70 (1H, bs, NH_2_), 7.27–7.06 (4H, m, H3′, H4′, H5′, H6′), 4.60 (2H, d, *J* = 4.9 Hz, CH_2_), 2.30 (3H, s, CH_3_). ^13^C-NMR (125 MHz, DMSO) δ 169.0, 154.4, 146.4, 137.2, 135.9, 130.3, 130.2, 127.5, 127.2, 126.7, 126.1, 41.8, 18.8. Elemental analysis found: C, 64.28%; H, 6.07%; N, 22.90%. Calculated for C_13_H_14_N_4_O (MW 242.28): C, 64.45%; H, 5.82%; N, 23.13%.

*3-[(4-Methoxybenzyl)amino]pyrazine-2-carboxamide* (**7**). Yellow solid. Yield 73%; M.p. 148.5–151.3 °C; IR (ATR-Ge, cm^−1^): 3464 (-NH-), 3126 (-CONH_2_), 2952 (-OCH_3_), 2910 (-CH_2_-), 1690 (-C=O), 1612, 1582, 1530, 1509, 1469, 1413, 1245, 1173 (arom.); ^1^H-NMR (300 MHz, DMSO) δ 9.04 (1H, t, *J* = 5.7 Hz, NH), 8.24 (1H, d, *J* = 2,4 Hz, H6), 8.15 (1H, bs, NH_2_), 7.78 (1H, d, *J* = 2,5 Hz, H5), 7.67 (1H, bs, NH_2_), 7.28–7.23 (2H, m, H2′,H6′), 6.90–6.85 (2H, m, H3′, H5′), 4.55 (2H, d, *J* = 5.7 Hz, CH_2_), 3.71 (3H, s, OCH_3_). ^13^C-NMR (75 MHz, DMSO) δ 168.88, 158.44, 154.25, 146.52, 131.45, 130.10, 128.82, 126.65, 114.00, 55.20, 43.05. Elemental analysis found: C, 60.74%; H, 5.93%; N, 21.71%. Calculated for C_13_H_14_N_4_O_2_ (MW 258.25): C, 60.45%; H, 5.46%; N, 21.69%. 

*3-[(4-Methylbenzyl)amino]pyrazine-2-carboxamide* (**8**). Yellow solid. Yield 72%; M.p. 145.0–146.9 °C; IR (ATR-Ge, cm^−1^): 3427 (-NH-), 3195 (-CONH_2_), 2926 (-CH_2_-), 1669 (-C=O), 1579, 1531, 1503, 1419, 1333, 1232, 1188 (arom.); ^1^H-NMR (300 MHz, DMSO) δ 9.08 (1H, t, *J* = 6.0 Hz, NH), 8.23 (1H, d, *J* = 2.4 Hz, H6), 8.16 (1H, s, NH_2_), 7.78 (1H, d, *J* = 2.5 Hz, H5), 7.68 (1H, s, NH_2_), 7.21–7.10 (4H, dd, H2′, H3′, H5′, H6′), 4.58 (2H, d, *J* = 5.8 Hz, CH_2_), 2.25 (3H, s, CH_3_). ^13^C-NMR (75 MHz, DMSO) δ 168.95, 154.30, 146.52, 136.54, 136.08, 130.15, 129.13, 127.40, 126.68, 43.32, 20.84. Elemental analysis found: C, 64.98%; H, 6.32%; N, 22.84%. Calculated for C_13_H_14_N_4_O (MW 242.28): C, 64.45%; H, 5.82%; N, 23.13%. 

*3-[(4-Aminobenzyl)amino]pyrazine-2-carboxamide* (**9**). Ochre solid. Yield 46%; M.p. 140.8–142.3 °C; IR (ATR-Ge, cm^−1^): 3426 (-NH-), 3324 (-NH_2_), 3194 (-CONH_2_), 1670 (-C=O), 1614, 1578, 1514, 1464, 1413, 1231, 1191 (arom.); ^1^H-NMR (300 MHz, DMSO) δ 8.89 (1H, t, *J* = 5.5 Hz, NH), 8.24 (1H, d, *J* = 2.3 Hz, H6), 8.13 (1H, s, NH_2_), 7.76 (1H, d, *J* = 2.3 Hz, H5), 7.65 (1H, s, NH_2_), 7.03–6.97 (2H, m, H2′, H6′), 6.54–6.48 (2H, m, H3′, H5′), 4.97 (2H, s, NH_2_′), 4.41 (2H, d, *J* = 5.5 Hz, CH_2_). ^13^C-NMR (75 MHz, DMSO) δ 168.97, 154.24, 147.91, 146.58, 129.87, 128.63, 126.49, 126.04, 114.03, 43.56. Elemental analysis found: C, 58.78%; H, 5.79%; N, 29.04%. Calculated for C_12_H_13_N_5_O (MW 243.26): C, 59.25%; H, 5.39%; N, 28.79%. 

*3-[(2-Chlorobenzyl)amino]pyrazine-2-carboxamide* (**10**). Ochre solid. Yield 80%; M.p. 182.4–184.3 °C; IR (ATR-Ge, cm^−1^): 3456 (-NH-), 3291 (-CONH_2_), 2921 (-CH_2_-), 1661 (-C=O), 1583, 1569, 1509, 1471, 1409, 1231, 1187 (arom.); ^1^H-NMR (300 MHz, DMSO) δ 9.19 (1H, t, *J* = 6.1 Hz, NH), 8.22 (1H, d, *J* = 2.4 Hz, H6), 8.19 (1H, s, NH_2_), 7.81 (1H, d, *J* = 2.3 Hz, H5), 7.71 (1H, s, NH_2_), 7.47–7.41 (1H, m, H5′), 7.36–7.22 (3H, m, H2′, H3′, H4′), 4.71 (2H, d, *J* = 6.1 Hz, CH_2_). ^13^C-NMR (75 MHz, DMSO) δ 168.86, 154.17, 146.44, 136.69, 132.43, 130.51, 129.40, 128.90, 128.77, 127.37, 126.96, 41.64. Elemental analysis found: C, 55.11%; H, 4.65%; N, 21.02%. Calculated for C_12_H_11_ClN_4_O (MW 262.69): C, 54.87%; H, 4.22%; N, 21.33%.

*3-[(2-Fluorobenzyl)amino]pyrazine-2-carboxamide* (**11**). Dark yellow solid. Yield 66%; M.p. 171.3–174.3 °C; IR (ATR-Ge, cm^−1^): 3446 (-NH-), 3277 (-CONH_2_), 2921 (-CH_2_-), 1666 (-C=O), 1572, 1534, 1514, 1486, 1420, 1225, 1190 (arom.); ^1^H-NMR (300 MHz, DMSO) δ 9.13 (1H, t, *J* = 5.9 Hz, NH), 8.23 (1H, d, *J* = 1.8 Hz, H6), 8.18 (1H, s, NH_2_), 7.80 (1H, d, *J* = 1.7 Hz, H5), 7.71 (1H, s, NH_2_), 7.40–7.08 (4H, m, H2′, H3′, H4′, H5′), 4.70 (2H, d, *J* = 6.0 Hz, CH_2_). ^13^C-NMR (75 MHz, DMSO) δ 168.88, 162.12, 158.88, 154.21, 146.45, 130.44, 129.57–128.54 (m), 126.91, 126.36 (d, *J* = 14.7 Hz), 124.53 (d, *J* = 3.4 Hz), 115.33 (d, *J* = 21.0 Hz), 37.62 (d, *J* = 4.4 Hz). Elemental analysis found: C, 58.73%; H, 4.19%; N, 22.32%. Calculated for C_12_H_11_FN_4_O (MW 246.24): C, 58.53%; H, 4.50%; N, 22.75%. 

*3-[(4-Trifluoromethylbenzyl)amino]pyrazine-2-carboxamide* (**12**). Yellow solid. Yield 46%; M.p. 136.8–138.3 °C; IR (ATR-Ge, cm^−1^): 3423 (-NH-), 3257 (-CONH_2_), 1678 (-C=O), 1584, 1532, 1510, 1414, 1231, 1189 (arom.), 1322 (-CF_3_); ^1^H-NMR (300 MHz, DMSO) δ 9.25 (1H, t, *J* = 6.1 Hz, NH), 8.20 (1H, d, *J* = 2.4 Hz, H6), 8.18 (1H, s, NH_2_), 7.80 (1H, d, *J* = 2.4 Hz, H5), 7.74 (1H, s, NH_2_), 7.65 (2H, d, *J* = 8.0 Hz, H2′, H6′), 7.51 (2H, d, *J* = 7.9 Hz, H3′. H5′), 4.74 (2H, d, *J* = 6.1 Hz, CH_2_). ^13^C-NMR (75 MHz, DMSO) δ 168.91, 154.23, 146.44, 145.00 (d, *J* = 1.7 Hz), 130.52, 127.86, 127.34, 126.97, 126.35, 125.38 (q, *J* = 3.8 Hz), 122.74, 43.14, 40.53. Elemental analysis found: C, 52.83%; H, 3.87%; N, 18.73%. Calculated for C_13_H_11_F_3_N_4_O (MW 296.25): C, 52.71%; H, 3.74%; N, 18.91%.

*3-[(2-Trifluoromethylbenzyl)amino]pyrazine-2-carboxamide* (**13**). Light yellow solid. Yield 55%; M.p. 173.7–175.3 °C; IR (ATR-Ge, cm^−1^): 3456 (-NH-), 3254 (-CONH_2_), 1672 (-C=O), 1586, 1578, 1516, 1414, 1232, 1189 (arom.), 1339 (-CF_3_); ^1^H-NMR (300 MHz, DMSO) δ 9.22 (1H, t, *J* = 5.9 Hz, NH), 8.21 (1H, d, *J* = 1.8 Hz, H6), 8.20 (1H, s, NH_2_), 7.82 (1H, d, *J* = 2.1 Hz, H5), 7.81 (1H, s, NH_2_), 7.72 (1H, *J* = 8.5 Hz, H5′), 7.64–7.39 (3H, m, H2′, H3′, H4′), 4.85 (2H, d, *J* = 5.9 Hz, CH_2_). ^13^C-NMR (75 MHz, DMSO) δ 168.88, 154.13, 146.46, 138.07 (d, *J* = 1.9 Hz), 132.90, 130.64, 128.78, 127.53, 127.04, 126.88–125.82 (m), 122.94, 40.36, 40.31. Elemental analysis found: C, 53.05%; H, 3.86%; N, 18.74%. Calculated for C_13_H_11_F_3_N_4_O (MW 296.25): C, 52.71%; H, 3.74%; N, 18.91%.

*3-[(2,4-Dimethoxybenzyl)amino]pyrazine-2-carboxamide* (**14**). Yellow solid. Yield 66%; M.p. 147.9–149.6 °C; IR (ATR-Ge, cm^−1^): 3403 (-NH-), 3215 (-CONH_2_), 2951 (-OCH_3_), 2832 (-CH_2_-), 1677 (-C=O), 1650, 1617, 1578, 1535, 1506, 1468, 1427, 1250, 1190, 1158 (arom.); ^1^H-NMR (300 MHz, DMSO) δ 9.00 (1H, t, *J* = 5.7 Hz, NH), 8.23 (1H, d, *J* = 2.4 Hz, H6), 8.12 (1H, s, NH_2_), 7.75 (1H, d, *J* = 2.4 Hz, H5), 7.64 (1H, s, NH_2_), 7.11 (1H, d, *J* = 8.3 Hz, H2′), 6.57 (1H, d, *J* = 2.4 Hz, H3′), 6.44 (1H, dd, *J* = 8.3, 2.4 Hz, H5′), 4.49 (2H, d, *J* = 5.7 Hz, CH_2_), 3.80 (3H, s, OCH_3_), 3.72 (3H, s, OCH_3_). ^13^C-NMR (75 MHz, DMSO) δ 189.90, 168.94, 160.05, 158.31, 154.34, 146.53, 129.85, 129.28, 126.64, 119.01, 104.51, 98.66, 55.61, 55.35. Elemental analysis found: C, 58.52%; H, 6.08%; N, 19.38%. Calculated for C_14_H_16_N_4_O_3_ (MW 288.30): C, 58.32%; H, 5.59%; N, 19.43%. 

*3-[(3-Nitrobenzyl)amino]pyrazine-2-carboxamide* (**15**). Light brown solid. Yield 26%; M.p. 165.6–168.3 °C; IR (ATR-Ge, cm^−1^): 3423 (-NH-), 3193 (-CONH_2_), 2923 (-CH_2_-), 1668 (-C=O), 1578, 1511, 1461, 1412, 1232, 1185 (arom.), 1522, 1347 (-NO_2_); ^1^H- NMR (300 MHz, DMSO) δ 9.28 (1H, t, *J* = 6.2 Hz, NH), 8.21 (1H, d, *J* = 2.5 Hz, H6), 8.19 (1H, s, NH_2_), 8.16 (1H, t, *J* = 2.0 Hz, H6′), 8.07 (1H, m, H4′), 7.81 (1H, d, *J* = 2.4 Hz, H5), 7.79 (1H, s, NH_2_), 7.77–7.70 (1H, m, H2′), 7.59 (1H, t, *J* = 7.9 Hz, H3′), 4.78 (2H, d, *J* = 6.2 Hz, CH_2_). ^13^C-NMR (75 MHz, DMSO) δ 168.82, 154.10, 147.99, 146.39, 142.62, 134.10, 130.64, 130.00, 127.05, 121.88, 121.84, 42.90. Elemental analysis found: C, 53.02%; H, 4.45%; N, 25.64%. Calculated for C_12_H_11_N_5_O_3_ (MW 273.25): C, 52.75%; H, 4.06%; N, 25.63%.

### 3.4. Biological Screening

#### 3.4.1. Antimycobacterial Evaluation

Antimycobacterial screening was performed against *M. tuberculosis* H37R_v_ CNCTC My 331/88, *M. kansasii* Hauduroy CNCTC My 235/80, and *M. avium* CNCTC My 152/73 (Czech National Collection of Type Cultures, National Institute of Public Health, Prague, Czech Republic) using isoniazid and pyrazinamide (Sigma-Aldrich) as standards. Culturing medium used for assays was Sula’s semisynthetic broth with adjusted pH 5.6 (Trios, Prague, Czech Republic). Cultures were grown at 37 °C in the dark and in a humid atmosphere. Tested compounds were dissolved in dimethyl sulfoxide (DMSO) and diluted with medium to final concentrations 100, 50, 25, 12.5, 6.25, 3.13 and 1.56 μg/mL except for INH where the dilution continues up to 0.1 μg/mL. The method used for this screening was a microdilution broth panel method. Mycobacterial inoculum was suspended in sterile water and the density was adjusted in the range between 0.5 and 1.0 McFarland scale. Suspensions were diluted with broth by 10^−1^, added to microtitration plates and incubated for 14 days for *M. tuberculosis*, 5–7 days for *M. kansasii* and 5 days for *M. avium*. Drug-free controls were included containing broth with DMSO. The final concentration of DMSO was 0.5% (*v*/*v*) and did not affect the growth of mycobacteria. Antimycobacterial activity was determined using a resazurin-based dye (Alamar blue) and results were read after 24 h of incubation after addition of 30 µL of stain (0.005% solution of resazurin sodium salt in the 1:1 mixture of water and 10% (*v*/*v*) water solution of Tween 80) [43]. Minimum inhibitory concentration was defined as the lowest concentration of tested compound which prevented colour change (from blue to pink).

Resistant strains were obtained as clinical isolates of *M. tuberculosis* labelled 234/2005, 9449/2007, Praha 1, Praha 4 and Praha 131. Microdilution broth panel method was used to determine MIC. Tested compounds were dissolved in DMSO, diluted with Sula’s semisynthetic medium to get final concentrations ranged between 1 and 1000 μM. INH was used as a standard in a sterile water solution at a concentration ranging from 0.5 to 250 μM. Suspensions of mycobacterial strains were adjusted to density 1.0 according to McFarland scale. MIC was determined as the lowest concentration that inhibited the visual growth of mycobacteria after incubation (37 °C) for 14 resp. 21 days.

#### 3.4.2. Evaluation of Activity Against *Mycobacterium smegmatis*

The antimycobacterial assay was performed with the fast growing *Mycobacterium smegmatis* CCM 4622 (ATCC 607) from Czech Collection of Microorganisms (Brno, Czech Republic). A technique used for activity determination was microdilution broth panel method using 96-well microtitration plates. Culturing medium was Middlebrook 7H9 broth (Sigma-Aldrich) enriched with 0.4% of glycerol (Sigma-Aldrich) and 10% of Middlebrook OADC growth supplement (Himedia, Mumbai, India). Tested compounds were dissolved in DMSO (Sigma-Aldrich) then Middlebrook broth was added to obtain concentration 2000 µg/mL. Standards used for activity determination were isoniazid (INH), rifampicin (RIF) and ciprofloxacin (CPX) (Sigma-Aldrich). Final concentrations were reached by binary dilution and addition of mycobacterial suspension and were set as 500, 250, 125, 62.5, 31.25, 15.63, 7.81 and 3.91 µg/mL except to standards ciprofloxacin and rifampicin where the final concentrations were 12.5, 6.25, 3.13, 1.56, 0.78, 0.39, 0.20 and 0.10 µg/mL. Drug-free controls were included containing Middlebrook 7H9 broth and DMSO. The final concentration of DMSO did not exceeded 2.5% (*v*/*v*) and did not affect the growth of *M. smegmatis*. *Mycobacterium smegmatis* was cultured in liquid medium Middlebrook 7H9 enriched with glycerol (0.4%) and OADC growth supplement (10%). Bacteria were always passaged after seven days of incubation. Testing mycobacterial suspension was prepared by adjusting a density to 1.0 McFarland scale and diluting it with broth in a ratio of 1:20. Plates were sealed with a polyester adhesive film and incubated at 37 °C in the dark without agitation. Addition of 0.01% solution of resazurin sodium salt (20 μL) followed after 48 h of incubation. Stain was prepared by dissolving resazurin sodium salt (Sigma-Aldrich) in deionised water to get a 0.02% solution. Then 10% aqueous solution of Tween 80 (Sigma-Aldrich) was prepared. Both liquids were mixed up making use of the same volumes and filtered through a syringe membrane filter. After addition of dye, microtitration panels were then incubated for additional 4 h. Antimycobacterial activity was expressed as minimum inhibitory concentration (MIC) and a value was read on the basis of colour change (blue colour—active; pink colour—inactive). MIC values for standards were in ranges 7.81–15.63 µg/mL for INH, 0.78–1.56 µg/mL for RIF and 0.10–0.20 µg/mL for CPX. All experiments were conducted in duplicate.

#### 3.4.3. Antibacterial and Antifungal Activity Evaluation

This assay was based on the microdilution broth panel method in 96-well microtitration plates [44]. Organisms used in experiments included strains *Staphylococcus aureus* CCM 4516/08, *Escherichia coli* CCM 4517, *Pseudomonas aeruginosa* CCM 1961 (Czech Collection of Microorganisms, Brno, Czech Republic) that are recommended as standards for antibacterial screening. Additional strains were acquired from Department of Clinical Microbiology, University Hospital, Hradec Kralove, Czech Republic as clinical isolates: *Staphylococcus aureus* H 5996/08—methicillin resistant (MRSA), *Staphylococcus epidermidis* H 6966/08, *Enterococcus faecalis* J 14365/08, *Klebsiella pneumoniae* D 11750/08, *Klebsiella pneumoniae* J 14368/08—ESBL positive. All microorganisms were cultured on Mueller-Hinton agar (MHA) (Difco/Becton Dickinson, Detroit, MI, USA) at 35 °C. Bacterial inoculum was prepared by suspending a colony in sterile 0.85% saline. The cell density of the inoculum was adjusted to 0.5 McFarland scale. Compounds were dissolved in DMSO and antibacterial activity was determined in Mueller-Hinton broth (Difco/Becton Dickinson) buffered to pH 7.0. Negative controls (growth controls) were consisted of medium and DMSO. The final concentration of DMSO did not exceed 1% (*v*/*v*) of the total solution composition. Minimum inhibitory concentration (MIC) was defined as inhibition of bacterial growth compared to control and was determined after 24 and 48 h of static incubation at 35 °C. Bacitracin, neomycin, ciprofloxacin and phenoxymethylpenicillin were chosen as standards.

Antifungal screening was also carried out using microdilution broth method with tested strains—*Candida albicans* ATCC 44859, *C. tropicalis* 156, *C. krusei* E28, *C. glabrata* 20/I, *Trichosporon asahii* 1188, *Aspergillus fumigatus* 231, *Lichtheimia corymbifera* 272 and *Trichophyton mentagrophytes* 445. Compounds were dissolved in DMSO and diluted using RPMI 1640 medium with glutamine buffered to pH 7.0 (MOPS) in a twofold manner. The final concentration of DMSO in tested medium did not exceed 2.5% (*v*/*v*). Static incubation was performed at 35 °C in a humid atmosphere in the dark for 24 and 48 h (resp. 72 and 120 h for *Trichophyton mentagrophytes*). Drug-free controls were included and fluconazole, amphotericin B, voriconazole and nystatin were used as standards [45].

#### 3.4.4. Cytotoxicity Determination

Human hepatocellular liver carcinoma cell line HepG2 (passage 12) purchased from Health Protection Agency Culture Collections (ECACC, Salisbury, UK) was routinely cultured in Minimum Essential Eagle Medium (MEM, Sigma-Aldrich) supplemented with 10% fetal bovine serum (PAA Laboratories GmbH, Pasching, Austria), 1% L-glutamine solution (Sigma-Aldrich) and non-essential amino acid solution (Sigma-Aldrich) in a humid atmosphere containing 5% of CO_2_ at 37 °C. Cells were harvested after trypsin/EDTA (Sigma-Aldrich) treatment at 37 °C for subculturing. Cells treated with tested substances were used as experimental groups. Untreated HepG2 cells were applied to represent control groups. HepG2 cells were seeded in density 10,000 cells per well in a 96-well plates and were treated with tested substances dissolved in DMSO next day. The tested compounds were prepared in seven concentrations (1–1000 µM) in triplicates. The controls (100% cell viability, 0% cell viability, control without cells, and vehicle controls) were also prepared in triplicates. The reagent from CellTiter 96 Aqueous One Solution Cell Proliferation Assay (CellTiter 96, Promega Corporation, Madison, WI, USA) was added after incubation in a humidified atmosphere containing 5% of CO_2_ at 37 °C lasting for 24 h. Additional incubation at 37 °C followed and absorbance was recorded at 490 nm. A standard toxicological parameter IC_50_ was calculated using GraphPad Prism software 6.02 (GraphPad Software, La Jolla, CA, USA).

#### 3.4.5. Lipophilicity Determination

Theoretical lipophilicity parameters log*P* and Clog*P* were calculated by algorithms of program CS ChemBioDraw Ultra 14.0 (CambridgeSoft, Cambridge, MA, USA). Experimental lipophilicity parameter log*k* values were determined using HPLC method. Agilent Technologies 1200 SL liquid chromatography with SL G1315C diode-array detector, ZORBAX XDB-C18 5 μm, 4 × 4 mm, Part No. 7995118-504 chromatographic pre-column and ZORBAX Eclipse XDB-C18 5 μm, 4.6 × 250 mm, Part No. 7995118-585 column (Agilent Technologies Inc., Colorado Springs, CO, USA) were used in the HPLC system. The separation process was handled by Agilent ChemStation, version B.04.02 extended with spectral module (Agilent Technologies Inc.). A mixture of methanol (HPLC grade, 70%) and water (HPLC-Milli-Q Grade, 30%) was used as mobile phase. The total flow rate of the column was set to 1.0 mL/min, injection 20 μL, column temperature 30 °C. 210 nm as detection wavelength and 270 nm as monitor wavelength were chosen. A methanolic solution of KI was used for the determination of dead time (TD). Retention times (TR) of synthesized compounds were measured in minutes. Capacity factors *k* were calculated according to formula *k* = (TR − TD)/TD, where TR is the retention time of the solute and TD denotes the dead time obtained via an unretained analyte (KI). Log*k*, which was calculated from the capacity factor *k*, is used as the lipophilicity index.

### 3.5. Computational Studies

All molecular modelling was done using Schrödinger Suite (Release 2014-2) and visualizations were prepared in Maestro 9.8 (Schrödinger, LLC, New York, NY, USA). Docking was carried out using software Glide (Schrödinger). The crystal structure of enzyme enoyl-ACP reductase was prepared using PDB structure 4TZK as starting geometry, using Maestro Protein Preparation Wizard with default settings. The ligand and the non-bonding water molecules were removed. Restrain energy minimization was performed using OPLS-2005 force field (to gradient of 0.001 RMS kcal/mol/A^2^). Original ligand was used to determine a binding site using grid generation tool. The ligand structures were prepared in Maestro 2D sketcher. Compounds were subsequently docked using the standard precision (SP) protocol with flexible sampling of ligands and without any constraints. Top-scored compounds were subsequently docked using the extra precision (XP) protocol. The docking protocol was validated by re-docking the original co-crystalized ligand. Average RMSD for five best-scored poses after redocking of the original ligand was 0.85 Å. Conformational search for compound **14** was performed using MOE 2016.08 (Molecular Operating Environment, Chemical Computing Group Inc., Montreal, QC, Canada). 

## 4. Conclusions

Four of fifteen prepared compounds showed notable antimycobacterial activity against *M. tuberculosis* H37R_v_. Compounds **4**, **8**, **9** and **12** exerted activities similar to or better than PZA. 4‑Methylbenzylamino derivative **8** also showed activity nearly the same as that of the standard isoniazid. Prepared benzylaminopyrazine-2-carboxamides were compared to previously synthesized benzylaminopyrazines with different substitution patterns on the pyrazine ring. The corresponding compounds were determined to be active, nevertheless the title structures of this research without a carbonitrile moiety appeared to exert at least double activity, even though their lipophilicity was lower. Additional screening against resistant strains of *M. tbc* showed moderate activity for compounds **8** and **9**. The values were not comparable to substances with proven susceptibility. Cytotoxicity assays performed on HepG2 cells confirmed preceding assumption that molecules containing a trifluoromethyl moiety are cytotoxic despite the fact they are usually active. On the other hand, compounds **8** and **9** showed very low cytotoxicity and their calculated selectivity index was considered to be propitious. Antitubercular activity of these two compounds is rather specific than non-selective based on the cytotoxic effect. Docking studies did not exclude that inhibition of InhA might contribute to the mechanism of action of the presented derivatives. However, comparison of in vitro and in silico results suggests that InhA inhibition is probably not the main mechanism of action of these 2,3-disubstituted derivatives, or, the disagreement might be caused by differences in mycobacterial cell penetration among individual compounds. Nevertheless, predicted binding poses of active derivatives exhibit the key interactions observed with the most of the confirmed InhA inhibitors. The pyrazine carboxamide group plays a crucial role by forming the important interactions with Tyr 158 and NAD^+^. Substitution of the pyrazine core seems to determine the affinity of the assessed compounds and further stabilize the possible inhibitor within the active site. This enables interactions with various residues within the pocket and therefore anchor the inhibitor in the active site. Compounds **8** and **9** are suitable for further structural modifications that might improve their biological properties (activity, cytotoxicity) and physicochemical properties (solubility, lipophilicity).

## Figures and Tables

**Table 1:** Summary of prepared compounds, calculated and measured parameters of lipophilicity, results of antimycobacterial and antibacterial evaluation expressed as minimum inhibitory concentration (MIC) compared to standards.

No.	R	MW	Log*P*/Clog*P*	Log*k*	Minimum Inhibitory Concentrations			
					*M. tbc* H37R_v_ (µg/mL)	*M. tbc* H37R_v_ (µM)	*M. smegmatis* (µg/mL)	Bacteria (µM)
**1**	H	228.25	0.63/1.64	0.215	>100	n.a.	≥500	>500
**2**	3-Cl	262.69	1.19/2.36	0.373	>100	n.a.	≥500	>500
**3**	3,4-Cl	298.14	1.75/2.95	0.470	>100	n.a.	≥500	>500
**4**	3-CF_3_	297.14	1.56/2.53	0.403	12.5	42	250	31.25 SA ^a^
**5**	4-Cl	262.69	1.19/2.36	0.373	>100	n.a.	250	250 SA ^a^
**6**	2-CH_3_	242.28	1.12/2.09	0.320	>100	n.a.	≥500	>500
**7**	4-OCH_3_	258.25	0.51/1.56	0.194	>100	n.a.	≥500	>500
**8**	4-CH_3_	242.28	1.12/2.14	0.441	1.56	6	≥500	>500
**9**	4-NH_2_	243.26	−0.17/0.42	−0.313	6.25	26	250	>500
**10**	2-Cl	262.69	1.19/2.36	0.441	>100	n.a.	≥500	>500
**11**	2-F	246.24	0.79/1.79	0.248	>100	n.a.	≥500	125 EF ^b^
**12**	4-CF_3_	296.25	1.56/2.53	0.502	12.5	42	≥500	125 EF ^b^
**13**	2-CF_3_	296.25	1.56/2.53	0.495	>100	n.a.	≥500	125 EF ^b^
**14**	2,4-OCH_3_	288.30	0.38/1.65	0.274	>100	n.a.	≥500	62.5 EF ^b^
**15**	3-NO_2_	273.25	n.d./1.39	0.101	>100	n.a.	≥500	>500
**INH**	-	137.14	-	-	0.39	3	7.81–15.63	-
**PZA**	-	123.12	-	-	12.5	102	≥500	-
**RFM**	-	822.94	-	-	n.d.	n.d.	0.78–1.56	-
**CPX**	-	331.37	-	-	n.d.	n.d.	0.10–0.20	-

^a^ *Staphylococcus aureus* CCM 4516/08; ^b^ *Enterococcus faecalis* J 14365/08. Log*P*/Clog*P*—calc. parameter of lipophilicity (ChemDraw 15.0); Log*k*—experiment. determined lipophilicity; n.a.—not active; n.d.—not determined.

**Table 2:** Summary of prepared compounds, calculated and measured parameters of lipophilicity, results of antimycobacterial and antibacterial evaluation expressed as minimum inhibitory concentration (MIC) compared to standards.

	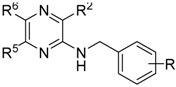				MIC against *M. tbc* H37R_v_	
No.	R	R^2^	R^5^	R^6^	µg/mL	µM
**1**	H	CONH_2_	H	H	>100	-
**1-A**	H	CONH_2_	CN	H	>100	-
**1-B**	H	CN	CN	H	12.5	78
**1-C**	H	CH_3_	CN	CN	25	100
**4**	3-CF_3_	CONH_2_	H	H	12.5	42
**4-A**	3-CF_3_	CONH_2_	CN	H	25	78
**4-B**	3-CF_3_	CN	CN	H	>100	-
**4-C**	3-CF_3_	CH_3_	CN	CN	12.5	39
**8**	4-CH_3_	CONH_2_	H	H	1.56	6
**8-A**	4-CH_3_	CONH_2_	CN	H	12.5	47
**8-B**	4-CH_3_	CN	CN	H	6.25	25
**8-C**	4-CH_3_	CH_3_	CN	CN	25	95
**9**	4-NH_2_	CONH_2_	H	H	6.25	26
**9-A**	4-NH_2_	CONH_2_	CN	H	12.5	47
**9-B**	4-NH_2_	CN	CN	H	25	100
**9-C**	4-NH_2_	CH_3_	CN	CN	25	95
**INH**	-	-	-	-	0.39	3
**PZA**	-	-	-	-	12.5	102

**Table 3:** Activity of two leading compounds **8** and **9** against resistant *Mycobacterium tuberculosis* strains presented as MIC values.

No.	R	Minimum inhibitory concentrations [µM]									
		*M. tbc* 9449/2007		*M. tbc* 234/2005		*M. tbc* PRAHA 1		*M. tbc* PRAHA 4		*M. tbc* PRAHA 131	
		14 days	21 days	14 days	21 days	14 days	21 days	14 days	21 days	14 days	21 days
**8**	4-CH_3_	250	250	250	250	250	250	250	250	250	250
**9**	4-NH_2_	62.5	62.5	62.5	62.5	62.5	62.5	62.5	62.5	62.5	125
**INH**	-	62.5	62.5	15.63	15.63	15.63	15.63	15.63	31.25	15.63	31.25
**RFM**	-	>15.63	>15.63	>15.63	>15.63	>15.63	>15.63	>15.63	>15.63	>15.63	>15.63

MDR-TB strains: 234/2005 resistant to INH, RFM, rifabutin, streptomycin, ethambutol and ofloxacin; Praha 1 resistant to INH, RFM, rifabutin, streptomycin, ethambutol and clofazimine; 9449/2007 and Praha 4 both resistant to INH, RIF, rifabutin, ethambutol and streptomycin; XDR-TB strain: Praha 131 resistant to INH, RIF, rifabutin, streptomycin, ethambutol, ofloxacin, gentamicin and amikacin.

**Table 4:** Cytotoxicity of active compounds and calculated selectivity index (SI) values.

Compound	IC_50_ (µM)	SI (IC_50_/MIC)
**4**	76.5	1.8
**8**	>250	>41.7
**9**	>750	>28.8
**12**	57.3	1.4

**Table 5:** Results of affinity calculations by Glide Scoring function in relation with in vitro activity against *M. tuberculosis* H37R_v_.

Compound	Affinity (kcal/mol)	MIC *M. tbc* H37R_v_ (µM)
**4TZK Ligand**	−9.82	-
**4**	−8.47	42
**8**	−7.40	6
**9**	−7.82	26
**12**	−8.49	42
